# A QALY loss is a QALY loss is a QALY loss: a note on independence of loss aversion from health states

**DOI:** 10.1007/s10198-018-1008-9

**Published:** 2018-09-18

**Authors:** Stefan A. Lipman, Werner B. F. Brouwer, Arthur E. Attema

**Affiliations:** Erasmus School of Health Policy & Management, Erasmus University, P.O. Box 1738, 3000 DR Rotterdam, The Netherlands

**Keywords:** Loss aversion, Prospect theory, QALY, Utility of life duration, Quality of life, Robustness, B41, D03, D81, I10

## Abstract

Evidence has accumulated documenting loss aversion for monetary and, recently, for health outcomes—meaning that, generally, losses carry more weight than equally sized gains. In the conventional Quality-Adjusted Life Year (QALY) models, which comprise utility for quality and length of life, loss aversion is not taken into account. When measuring elements of the QALY model, commonly, the (implicit) assumption is that utility for length and quality of life are independent. First attempts to quantify loss aversion for QALYs typically measured loss aversion in the context of life duration, keeping quality of life constant (or vice versa). However, given that QALYs are multi-attribute utilities, it may be possible that the degree of loss aversion is dependent on, or inseparable from, quality of life and non-constant. We test this assumption using non-parametric methodology to quantify loss aversion, under different levels of quality of life. We measure utility of life duration for four health states within subjects, and present the results of a robustness test of loss aversion within the QALY model. We find loss aversion coefficients to be stable at the aggregate level, albeit with considerable heterogeneity at the individual level. Implications for applied work on prospect theory within health economics are discussed.

## Introduction

Like other decisions, medical decisions often involve trade-offs between gains and losses in different domains. In health economics, an important trade-off concerns that between length and quality of life (QoL), also in the context of health state valuations. Research in behavioral economics and psychology has established that in such trade-off losses typically carry more weight than gains of the same size. This sensitivity to losses is referred to as *loss aversion* [1, 3]. Recently, scholars demonstrated the importance of loss aversion within the health domain, both for life duration [4–7] and quality of life (QoL) [7–9]. In health economic analyses, utilities are often defined as a product of these two attributes, jointly comprising Quality-Adjusted Life Years (QALYs) [10]. Commonly, the utility function over these two outcomes is decomposed into separate utility functions over life duration and QoL. This separability of QALYs is, however, only possible under several assumptions, which have solely been tested under conditions in which no distinction is made between gains and losses [11].

Here, we use prospect theory (PT), which incorporates loss aversion and judges changes from the perspective of some relevant reference point (RP). Bleichrodt and colleagues [11] established that, when considering multi-attribute outcomes, such as QALYs, gains and losses may be determined per attribute with separate attribute-specific RPs. This also makes it possible quantify loss aversion, to see how much more weight losses carry than gains. Earlier attempts at quantifying loss aversion under PT have typically focused on single attributes within the QALY framework, for example by obtaining loss aversion for life duration while maintaining QoL constant [4, 5] or vice versa [8]. Although these studies produced similar median estimates of loss aversion, with health losses receiving between 1.5 and 2 times more weight than gains, they did not allude to the issue of separability. In other words, these studies ignored the possibility that loss aversion for one attribute (e.g., length of life) depends on the level of the other attribute (which is typically held constant) and, hence, assumes loss aversion for health outcomes to be constant, independent of their QALY profile.

However, it could be the case that some QALY losses carry more weight relative to commensurate QALY gains than others, for example if loss aversion is more pronounced for more severe health states. In this article, we test this assumption using a non-parametric method [12] to quantify loss aversion over life duration, under varying levels of QoL. This non-parametric method was developed recently and allows the estimation of utility curvature and loss aversion without imposing parametric assumptions on either. Earlier work has argued that the choice of parametric family or functional form restricts interpretation of subjects’ choice patterns, and may lead to considerable bias especially for extreme cases [12, 13]. This method has been adapted to and used in the health domain before [5].

## Theoretical framework

Consider a decision maker facing choices with regard to his health under uncertain conditions, operationalized by presenting decision makers with risky prospects representing different life durations and QoL. We assume completeness and monotonicity for both attributes. We consider lotteries involving chronic health profiles, described as $(\beta ,T)$, where *β* represents QoL and *T* duration in years. According to the generalized QALY model [14], a decision maker’s preferences for health profiles can be represented by the following:

1 $$V(\beta ,T)=U(\beta ) \times L(T),$$

with $V(\beta ,T)$ being a product of *U*(*β*), the utility of *β*, and *L*(*T*) denoting the utility of *T* life years.

Here, we assume PT under risk with a sign-dependent utility function for life duration, so that gains are evaluated differently than losses, relative to an attribute-specific RP. We assume that, through instruction, it is possible to set this attribute-specific RP to a specific health condition ${\beta _{\text{c}}}$ and life duration ${T_0}$. To elicit a continuous utility function for life duration, we elicit a standard sequence for life duration that runs through $L({T_0})=0$. Meanwhile, we keep QoL constant at ${\beta _{\text{c}}}$ throughout the task. We repeat this process under different levels of ${\beta _{\text{c}}}$.

We elicit the utility function for life duration, relative to this RP, both for gains and losses for the different health states. Hence, we obtain ${L^i}(T)$ for each ${\beta _{\text{c}}}$, with $i=+$ for gains and $i= -$ for losses. ${L^i}(T)$ is a standard ratio scale utility function, which is strictly increasing and real-valued with ${L^i}({T_0})=0$. We incorporate loss aversion by taking ${L^ - }(T)=\lambda$*L*(*T*) for *T* < ${T_0}$, where *λ* denotes a loss aversion index, with *λ* > 1 [= 1, < 1] indicating loss aversion [loss neutrality, gain seeking]. Hence, by obtaining the utility around the RP, the degree of loss aversion can be derived.

## Methods

A total of 111 students (average age 20.23, SD = 1.52) of Rotterdam School of Management (61 female) participated in this study for a course credit reward. Experimental sessions lasted for 25 min and were run with up to four subjects per session. One experimenter was presented in the room to answer questions. The experiment was computerized with Matlab.

To test the robustness of loss aversion, we used the non-parametric method [12] under four levels of QoL. In other words, each subject completed the non-parametric method four times, with a different ${\beta }_{c}$ throughout each of these four phases. This process allows us to obtain estimates of utility curvature and loss aversion for each of the four levels of QoL, and compare them within subjects.

QoL was defined by means of EQ-5D-5L health state descriptions [15], which utilize five domains: mobility, self-care, usual activities, pain/discomfort, and anxiety/depression. The 5L version of the EQ-5D distinguishes five levels of severity on each domain, ranging from ‘no problems’ to ‘extreme problems/unable to’. Health states are typically denoted by 5 digit codes like 22113, with each number representing severity of the relevant domain level of QoL. In this study, we used four relatively mild-to-moderate health states as RP ${\beta _{\text{c}}}$ in the non-parametric method: 11111, 21211, 31221, and 32341 (see “Appendix 1” for exact description). This was done to have variation in health states but avoid states worse than dead, for which no separate procedure was included.

The non-parametric method used here consisted of three stages which are described in detail in “Appendix 2”.^1^ The first stage connects the utility for gains and losses. The second and third stages employ the trade-off method developed by [16] to measure a standard sequence of outcomes in life years for gains $(x_{1}^{+},x_{2}^{+}, \ldots ,x_{5}^{+})$, and for losses $(x_{1}^{ - },x_{2}^{ - }, \ldots ,x_{5}^{ - })$. This enables measuring loss aversion, without imposing parametric assumptions on utility curvature.^2^ In addition, the standard sequences allow the testing of utility independence [11]. The three stages had slightly different instructions, providing context for the required trade-offs. The instructions were similar to those used by Lipman and colleagues [5]. During all the stages of the experiment, it was made clear to subjects that they should imagine living until 70 years in ${\beta _{\text{c}}}$, after which they would contract a disease, resulting in immediate death without any pain. Subjects completed a series of binary choices between two drugs which could change their situation (leading to gains and losses compared to living until 70). Employing a bi-section choice method, we obtained indifferences, set equal to the midpoint after the fifth binary choice. Some stimuli and constants relevant to the non-parametric method had to be set beforehand; these are listed in “Appendix 1”.

## Results

Seven subjects were excluded from further analyses for the following reasons: mechanical failure (*n* = 2), refusing to incur life year losses (*n* = 3), and observed misbehavior (e.g., rushing through the task, *n* = 2*)*. The results are reported for the reduced sample (*n* = 104).^3^ Throughout, we will first report aggregate analyses, where median parameters are compared for the whole sample, and refer to these as results at ‘the aggregate level’. Second, we will investigate individual results more closely, by classifying each individual according to classification rules reported in “Box 1” and we explore within-subjects parameter instability. We refer to these analyses as ‘individual-level analyses’.

Table 1 demonstrates the results at the aggregate level, by comparing point-estimates for utility curvature and loss aversion for each health state. We compared differences between health states using omnibus tests (i.e., comparing all four health states simultaneously), more specifically Friedman’s tests, which are robust against the violations of normality typically observed for parameters under the definitions reported in “Box 1”. Next, we compared all health states in pairs with Wilcoxon signed-rank tests. For the omnibus tests, no significant differences were observed between health states, both for utility curvature and loss aversion (all *p*’s > 0.06). When comparing parameter estimates in pairs of health states, some significant differences were observed. For loss aversion under both definitions, parameter estimates for β2 were significantly lower than for β3 (*p*’s < 0.03). All other pairwise comparisons for loss aversion yielded no significant differences (all *p*’s > 0.07). Using pairwise comparisons for utility curvature, we observe no significant differences for both parametric and non-parametric estimations (all *p*’s > 0.05).

**Table 1:** Median (IQR in brackets) parameter point-estimates for loss aversion under two definitions and utility curvature as defined by area under the curve (AUC) and power utility

Health state	β0: 11111	β1: 21211	β2: 31221	β3: 32341
Utility curvature				
AUC—gains	0.51 (0.42–0.63)	0.49 (0.38–0.59)	0.53 (0.44–0.64)	0.52 (0.41–0.70)
AUC—losses	0.51 (0.46–0.57)	0.50 (0.45–0.57)	0.50 (0.42–0.58)	0.49 (0.40–0.60)
Power—gains	0.96 (0.58–1.37)	1.07 (0.69–1.71)	0.91 (0.57–1.28)	0.78 (0.45–1.41)
Power—losses	0.93 (0.74–1.16)	0.94 (0.73–1.20)	0.97 (0.73–1.41)	1.02 (0.66–1.40)
Loss aversion				
Köbberling Wakker	1.97 (1.33–4.43)	1.93 (1.45–3.67)	1.88 (1.39–3.30)	2.13 (1.15–8.38)
Kahneman Tversky	2.13 (1.24–4.39)	1.94 (1.26–4.62)	2.10 (1.25–3.23)	2.51 (1.18–6.24)

In general, we observe close to linear utility for all health states, both for gains and losses.^4^ Furthermore, we observe considerable loss aversion at the aggregate level, with *λ* significantly greater than 1 for all ${\beta _{\text{c}}}$ (Wilcoxon tests: *p* < 0.001 for all *β*’s).

Table 2 demonstrates how subjects classify under different estimations of utility curvature and loss aversion (see “Box 1”). For all individual classifications, we observed that the conventionally assumed loss neutrality and linear utility curvature are not present in our data. Although, at the aggregate level, linear utility was found, when classifying individually, considerable heterogeneity in utility curvature was observed, with proportions of concave/convexity varying between definitions and health states. This finding could be explained by the near equal division of concavity/convexity in our sample, resulting in roughly linear utility at the aggregate level. For loss aversion, however, such an equal division was not visible, with the majority of subjects classifying as loss averse across definitions and health states.

**Table 2:** Individual classifications for utility curvature (*n* = concave, linear, convex) and loss aversion (*n* = loss averse, loss neutral, and gain seeking)

Health state	β0: 11111	β1: 21211	β2: 31221	β3: 32341
Utility curvature				
AUC—gains	55, 0, 49	47, 0, 54	61, 0, 43	61, 0, 43
AUC—losses	44, 0, 60	42, 0, 62	49, 0, 55	56, 0, 48
Power—gains	54, 0, 50	47, 0, 57	62, 0, 42	65, 0, 39
Power—losses	41, 0, 63	41, 0, 63	51, 0, 53	53, 0, 51
Loss aversion				
Köbberling/Wakker [2]	90, 0, 14	92, 0, 12	95, 0, 9	89, 0, 15
Kahneman/Tversky [1]	86, 0, 15	85, 0, 17	89, 0, 13	82, 0, 18

Our design allowed exploring point-estimate stability for utility curvature and loss aversion between different levels of ${\beta _{\text{c}}}$. To this end, we calculated the difference between the smallest and largest estimates within subjects (e.g., the lowest and highest $~\lambda$). Furthermore, to allude to within-subjects heterogeneity in classification, we calculated the proportion of subjects for whom classifications were dependent on health states (e.g., loss averse for β0–2 and gain seeking for β3). Both exploratory measures of within-subjects parameter and classification variance demonstrated considerable heterogeneity between health states (see Table 3). Finally, we investigated whether systematic patterns in utility curvature or loss aversion could be observed in our sample. To this end, we determined the extent to which subjects showed monotonically increasing (or decreasing) parameters (see Table 3). For loss aversion, this classification indicated that subjects became more (less) loss averse for increasing health state severity for $~{\beta _{\text{c}}}$. These analyses indicate that these patterns did occur, but only for a small part of our sample, again suggesting non-systematic heterogeneity of parameter estimates.

**Table 3:** Exploration of within-subjects heterogeneity for different health states

	Parameter point-estimate difference (max − min)	Health state-dependent classifications (%)	Parameters monotonically increasing/decreasing (%)
Utility curvature			
AUC—gains	0.26	75%	6% / 7%
AUC—losses	0.18	76%	4% / 7%
Power—gains	1.17	73%	7% / 7%
Power—losses	0.75	79%	5% / 5%
Loss aversion			
Köbberling Wakker [2]	5.10	37%	8% / 7%
Kahneman Tversky [1]	4.17	49%	6% / 7%

## Discussion

In this paper, we compared estimates for utility curvature and loss aversion for QALY outcomes under four levels of QoL, to test the robustness of these estimates. An extensive literature exists testing the validity of QALY models, which has documented mixed evidence with regard to the separability of life duration and QoL [e.g., 18–21]. In addition, many authors have investigated utility independence with regard to health state valuation (e.g., the relation between utilities and time horizon in the standard gambles), finding many descriptive violations of this independence [for a review, see: 20]. Ours was the first experimental test of this separability for QALY gains and losses separately, and we also tested the robustness of loss aversion. Our results, at the aggregate level, provided evidence that estimations of loss aversion and utility curvature are independent of QoL. However, loss aversion and utility curvature estimates were heterogeneous at the individual level, i.e., varied considerably between health states for the same individual.

Our findings are in many regards similar to earlier work that measured PT for QALY outcomes. We observed considerable loss aversion (defined over length of life), as was found in similar magnitude in earlier work applying similar methodology [5, 22], or with different elicitation methods [4, 8]. In contrast to what was observed in earlier applications of the non-parametric method for health outcomes [5, 22], we found linear utility for both gains and losses at the aggregate level. Applying a parametric approach to our non-parametric measurements did not affect these conclusions. However, when estimating individual classifications, we found none for whom our data supported this linearity, as we observed a near equal spread in concave/convex utility (i.e., averaging out to linear).

We document considerable heterogeneity in parameter estimates between subjects, and also observed such heterogeneity within subjects for different health states. Our exploratory analyses did not uncover systematic or monotonic patterns in this within-subjects heterogeneity. An explanation related to our chosen chained utility elicitation method could be that these individual differences occurred as a result of preference imprecision [23]. Such ‘noisy preferences’ could result in error propagation, i.e., cascading of errors or imprecision in the early stages of our chained method into later stages, producing differences in parameters between health states when errors occur randomly. Although earlier work using similar methodology [5, 24] observed no effects of error propagation, we cannot rule out it affected current results. Another factor contributing to possible error propagation in our study could be that we opted to obtain indifferences via bi-section only (to reduce complexity), whereas earlier work [5, 12] using this method applied a slider to obtain indifferences, allowing subjects to correct errors adaptively. Future work could explore this further, for example by adding a slider to obtain indifference points, using non-chained methodology, or running an error propagation simulation.

Some additional limitations of this study deserve noting. First, since this study involved a first test of independence of loss aversion in health, we used a convenience sample consisting of students. Of course, future extensions preferably should include representative samples to generalize our findings. Although power analyses suggested that our sample was adequately powered to detect small effects, using a larger sample could, perhaps, result in the detection of smaller effects, also given the large heterogeneity for parameter estimates reported here. Second, we assumed that it is possible to set the RP through instruction, while it may be the case that respondents took another RP in mind. Still, given the high loss aversion coefficients that we found, it seems plausible that our respondents, indeed, held the induced RP in mind. Finally, our study used four mild-to-moderate health states, including perfect health, while the EQ-5D descriptive system enables many more possible health states, with more severe health problems than our selection. Given the aim of our study, this is a clear limitation, as, perhaps, these states where insufficiently spaced in terms of utility for us to observe systematic patterns in loss aversion or utility curvature parameters. However, our empirical approach required us to make a fundamental assumption: monotonicity. The non-parametric method breaks down if monotonicity is not satisfied, i.e., if subjects prefer to lose years of life instead of gaining them. For more severe health states, monotonicity need not always hold [25]. Obviously, many other mild health states were available for our purposes, but to reduce cognitive strain for our subjects that we decided on including just four. For reference, these four health profiles receive utility weights ranging from 1.00 to 0.46 in the Dutch tariff [26], which we considered to be sufficient for our purposes. Future work could replicate our findings with a different or larger selection of health states.

Our findings may have implications for policy makers and researchers aiming to apply PT measurements to health-related decision-making. Our results imply that median parameters in applications of PT may have merit, as these estimates appear to be robust across different scenarios (in terms of QoL). For example, our work warrants the conclusion that, at the aggregate level, life year losses are weighed twice as much as similarly sized gains, regardless of QoL level. However, as our exploratory analyses of within-subject heterogeneity demonstrated, individuals’ loss aversion and utility curvature may depend on the health state used during elicitation. This heterogeneity at the individual level may be problematic for approaches using averages, like median-optimized parameters (e.g., [27]). When aiming to address PT biases for QALYs [28], such as loss aversion, at the individual level, our data would suggest that assuming such median loss aversion parameters may misrepresent individuals’ actual preferences and trade-offs. When one aims to apply PT to allude to biases in individual cases (e.g., in health state valuation), an individual approach may be more suitable, given both the considerable between subjects and between-health states’ heterogeneity reported in this study. Such corrections with individually estimated parameters could be too time-consuming and labor-intensive when applied separately for each economic evaluation. However, in many countries, such as the UK, QALYs are not derived individually, but from indirect preference-based classification systems, such as EQ5D or SF6D via social tariff lists [29]. Recent developments in de-biasing QALY measurement [5] suggest that it may be suitable and possible to apply the correction for PT at the individual level to obtain value sets for these social tariffs [see 30].^5^ When considering such individual correction, however, it seems important to consider which health state is used to quantify PT parameters.

In conclusion, although we observed large heterogeneity of loss aversion and utility of life duration depending on QoL, we failed to observe systematic patterns in this dependence, and observed no differences on average. Future work should aim to address whether this heterogeneity is method-dependent or due to systematic differences between individuals or health states. For now, it appears that, on average, loss aversion is equal across health states, i.e., a QALY loss is a QALY loss is a QALY loss, and it receives approximately twice as much weight as equally sized QALY gains.

## Appendix 1: Health states used in experiment

See Table 4 for more elaborate information on the health states that were utilized in this study.

**Table 4:** Health state descriptions based on EQ-5D-5L

Health state	β0: 11111	β1: 21211	β2: 31221	β3: 32341
Dutch Tariff [26]	1.00	0.88	0.79	0.46
You have … problems with walking	No	Slight	Moderate	Moderate
You have … problems with washing and dressing yourself	No	No	No	Slight
You have … problems with washing and dressing yourself	No	Slight	Slight	Moderate
… pain or discomfort	No	No	Slight	Severe
… anxious or depressed	Not	Not	Not	Not

## Appendix 2: Description of experimental method (adapted from Lipman et al. [5])

### Introduction and framing

Subjects were asked to imagine that they would live until 70 years in a health state denoted as health state C. This health state C would be varied for each repetition (4 in total) of the non-parametric method (i.e., ${\beta _{\text{c}}}$). After becoming 70, they were instructed that they would contract a deadly disease, which would lead to a direct, painless death. Their task was to compare two drugs and indicate their preferences between treatments given their health state C and the treatment options, which could be risky, or involve possible side-effects (i.e., losses of life).

### Stages of non-parametric method

The non-parametric method is chained, i.e., answers from the previous stage carry over to the next, meaning that differences in questions may exist between subjects. For a completely general description of the method, we refer to Abdellaoui and colleagues [12]. Throughout, as is common for applications of the trade-off method [16], any risky gamble had 50% chance (*p* = 0.5) of success. We denote such gambles as ${X_{\text{p}}}Y$, meaning that *X* is obtained with probability *p*, and *Y* otherwise. In our adaptation of the non-parametric method, outcomes (i.e., *X* and *Y*) reflected life years. Importantly, it was emphasized throughout that any life years gained or lost were to be spent in health state C. All indifferences were obtained via bi-section. Whenever a variable was elicited, a starting level had to be set to start the bi-section method. We chose to set it, such that the expected value would be equal for both treatments that subjects could choose from. For example, when eliciting the indifference $~Z\sim {10_{\text{p}}}0$, we would start at $Z$ = 5. This experiment was completely counterbalanced, meaning that health state order and gain–loss order were randomized between subjects. All pre-specified stimuli and elicited indifferences can be found in Table 5.

**Table 5:** Stimuli used in the three-stage procedure of the non-parametric method

	Variables elicited	Indifference	Implication	Stimuli
Stage 1	$L$	${G_{\text{p}}}L\sim {T_0}$	$U(x_{1}^{+})= - U(x_{1}^{ - })$	$G=5\;{\text{~years}}$ $p=$ 0.5 ${T_0}=70\;{\text{years}}$
	$x_{1}^{+}$	$x_{1}^{+}\sim {G_{\text{p}}}{T_0}$		
	$x_{1}^{ - }$	$x_{1}^{ - }\sim {L_{\text{p}}}{T_0}$		
Stage 2	$\mathcal{L}$	$x_{j}^{ + } \!_{{\text{p}}} {\mathcal{L}}\sim x_{{(j - 1)}}^{ + } \!_{{\text{p}}} \ell$	$U(x_{j}^{+}) - U(x_{{(j - 1)}}^{+})=U(x_{1}^{+}) - U(0)$	$\ell = - 1\;{\text{~year}}$ $j=5$
	$x_{j}^{+}$	$x_{j}^{+}{\!_{\text{p}}}\mathcal{L}\sim x_{{(j - 1)}}^{+}{\!_{\text{p}}}\ell$		
Stage 3	$\mathcal{G}$	${\mathcal{G}_{\text{p}}}x_{1}^{ - }\sim {\fancyscript{g}_{\text{p}}}{T_0}$	$U(x_{j}^{ - }) - U(x_{{(j - 1)}}^{ - })=U(x_{1}^{ - }) - U(0)$	$\fancyscript{g} =1\;{\text{~year}}$
	$x_{j}^{ - }$	${\mathcal{G}_{\text{p}}}x_{j}^{ - }\sim {\fancyscript{g}_{\text{p}}}x_{{(j - 1)}}^{ - }$		$j=5$

#### Stage 1: Connecting gains and losses

Subjects first faced a mixed gamble, which could increase their length of life by $~G$ years with probability *p*, or otherwise decrease it by $L$ years. They could also choose to take a drug that gave 0 years. The negative outcome $L~$was elicited by obtaining the following indifference ${G_{\text{p}}}L\sim {T_0}$, where ${T_0}$ indicates living until 70 in state C. As can be seen from Table 5, *G* was fixed at 5, while *L* was initially set at 2.5 and varied based on individual choices.

Next, two certainty equivalents (CEs) were elicited, which would form the starting points of the standard sequences elicited in stages 2 and 3. The CE for gains, i.e., the starting point for stage 2 was elicited by offering subjects a choice between a certain gain $x_{1}^{+}$ in life years (in state C), and a gamble offering $G$ (i.e., 5 years) with probability *p*, and 0 years otherwise. The amount of life years gained by taking the certain drug $(x_{1}^{+})$ was varied to obtain indifference $x_{1}^{+}\sim {G_{\text{p}}}{T_0}$. For losses, this procedure was exactly the same, i.e., subjects were offered a choice between a certain drug resulting in a loss of $x_{1}^{ - }$ life years in state C, and a risky drug. To introduce the loss domain, we instructed them that they had contracted another fatal disease that should also be treated, and thus explained their likely loss compared to ${T_0}$ (i.e., 70 years in C). We thus elicited $x_{1}^{ - }\sim {L_{\text{p}}}{T_0}$, providing the starting point ($x_{1}^{ - }$) for eliciting utility for losses in stage 3.

#### Stages 2 & 3: Trade-off method to elicit utility for gains and losses

The trade-off method consists of comparisons between two lotteries. Within our framing, this consisted of two risky drugs, which could increase subjects’ life duration in state C to a different extent. In addition, both drugs could have risks of adverse effects, and thus decrease lifetime in state C. To introduce the loss domain, subjects were instructed that they had contracted another fatal disease for which treatment was required. Subjects were instructed that they would compare a series of drugs to each other. This series constituted the procedure to elicit the standard sequence, which consists of a sequence of outcomes equally spaced in terms of utility (see [16] for proof).

Stage 2, i.e., the trade-off method for gains, commenced by us setting $\ell$, a small offset-loss of 1 year in state C. Subjects were offered a choice between two risky drugs: one would offer $x_{1}^{+}{\!_{\text{p}}}\mathcal{L}$, where $\mathcal{L}$ is a larger offset-loss which we aimed to elicit, while the other would offer ${\ell _p}{T_0}$. We varied $\mathcal{L}~t$o obtain the indifference $x_{1}^{+}{\!_{\text{p}}}\mathcal{L}\sim {\ell _{\text{p}}}{T_0}$. Next, we elicited the standard sequence $(x_{2}^{+}, \ldots ,x_{5}^{+})$ by eliciting indifferences in the form of $x_{j}^{+}\!{_{\text{p}}}\mathcal{L}\sim x_{{(j - 1)}}^{+}\!{_{\text{p}}}\ell$.

Stage 3, i.e., the trade-off method for losses, commenced by us setting $\fancyscript{g}$, a small offset-gain of 1 year in state C. Subjects were offered a choice between two risky drugs: one would offer ${\mathcal{G}_{\text{p}}}x_{1}^{ - }$ where $\mathcal{G}~$ is a larger offset-gain which we aimed to elicit, while the other would offer ${\fancyscript{g}_{\text{p}}}{T_0}$. We varied $\mathcal{G}~t$o obtain the indifference ${\mathcal{G}_{\text{p}}}x_{1}^{ - }\sim {\fancyscript{g}_{\text{p}}}{T_0}$. Next, we elicited the standard sequence $(x_{1}^{ - },x_{2}^{ - }, \ldots ,x_{5}^{ - })$ by eliciting indifferences in the form of ${\mathcal{G}_{\text{p}}}x_{j}^{ - }\sim {\fancyscript{g}_{\text{p}}}x_{{(j - 1)}}^{ - }$.

Repeating this procedure four times—for each health state (see Table 4)—resulted in four utility curves, and allowed us to obtain loss aversion parameters and both parametric and non-parametric estimates of utility curvature (see “Box 1”).

Box 1: Analyses of utility curvature and loss aversion We non-parametrically calculated the area under the curve for ${L^i}(T)$, which was normalized to $[0,1]$, for gains and $[0, - 1]{\text{~}}$ for losses. If utility is linear, the area under this normalized curve equals one-half for both gains and losses. Utility for gains in life duration is convex (concave) if the area under the curve is smaller (larger) than one-half, while, for losses, the opposite direction holds (convex > ½, concave < ½). Second, we fitted a parametric utility curve to our data by employing the power family, with the utility of life duration defined as ${x^\alpha }$ with $\alpha>0$. As is well known, for gains [losses] $\alpha>1$ corresponds to convex [concave] utility, $\alpha =1$ corresponds to linear utility, and $\alpha <1$ corresponds to concave [convex] utility. Kahneman and Tversky [1] defined loss aversion (λ) as $-U( - x)>U(x)$ for all $x>0$. To measure loss aversion coefficients according to this definition, we computed $-U( - x_{j}^{+})/U(x_{j}^{+})$ and $-U( - x_{j}^{ - })/U(x_{j}^{ - })~$ for $j=1, \ldots ,5$. As a result of the trade-off procedure, $U( - x_{j}^{+})$ and $U( - x_{j}^{ - })$ could usually not be observed directly and thus were determined through linear interpolation. Subjects were classified as loss averse if $-U( - x)/U(x)>1$ for more than half of the observations, as loss neutral if $-U( - x)/U(x)=1$ for more than half of the observations, and as gain seeking if $-U( - x)/U(x)<1$ for more than half of the observations. Köbberling and Wakker [2] provided an easier method to determine loss aversion. They defined loss aversion (*λ*) as the kink of utility at the reference point. That is, they defined loss aversion as $U_{ \uparrow }^{'}(0)/U_{ \downarrow }^{'}(0)$, with $U_{ \uparrow }^{'}(0)$ representing the left derivative and $U_{ \downarrow }^{'}(0)$ the right derivative of $U$ at the reference point. To operationalize this definition, we computed each subject’s coefficient of loss aversion as the ratio of $U(x_{1}^{ - })/x_{1}^{ - }$ over $U(x_{1}^{+})/x_{1}^{+}$, because $x_{1}^{ - }$ and $x_{1}^{+}$ are the loss and gain elicited closest to the reference point. A subject was classified as loss averse if $x_{1}^{+}/ - x_{1}^{ - }$ > 1, loss neutral if $x_{1}^{+}/ - x_{1}^{ - }$ = 1, and gain seeking if $x_{1}^{+}/ - x_{1}^{ - }$ < 1.

## Footnotes

1 For an elaborate, formal description of this method, see Abdellaoui and colleagues [12].

2 For more information on how utility curvature and loss aversion were determined, see “Box 1”.

3 The conventional post hoc power analyses suggested this sample was sufficiently powerful to enable detecting differences with at least small-effect sizes (Cohen’s *d* < 0.3), assuming *α* = 0.05 and statistical power at the recommended 80% level [17].

4 Wilcoxon tests comparing non-parametric curvature estimates with AUC 0.5, and parametric estimates with $\alpha =1$, produced no significant results for all β (all *p*’s > 0.08), with one exception: β1 power utility for gains, *p* = 0.04.

5 Although recent developments [5] suggest that it may be possible to de-bias QALYs at the individual level, several important questions with regard to the reliability of PT parameters and the validity of corrections based on these estimates remain unanswered. We believe that these warrant discussion before corrections based on PT are applied to correct value sets for social tariffs, as is discussed by [30].

## References

[1] Kahneman D, Tversky A (1979). Prospect theory: an analysis of decision under risk. J. Econom. Soc..

[2] Köbberling V, Wakker PP (2005). An index of loss aversion. J. Econ. Theory.

[3] Tversky A, Kahneman D (1992). Advances in prospect theory: cumulative representation of uncertainty. J Risk Uncertain..

[4] Attema AE, Brouwer WB, l’Haridon O (2013). Prospect theory in the health domain: a quantitative assessment. J. Health Econ..

[5] Lipman, S., Brouwer, W., Attema, A.E.: QALYs without Bias? Non-parametric correction of time trade-off and standard gamble weights based on prospect theory. SSRN (2017). https://ssrn.com/abstract=3051140 10.1002/hec.3895 PMC6618285 31237093

[6] Oliver A (2003). The internal consistency of the standard gamble: tests after adjusting for prospect theory. J. Health Econ..

[7] Bleichrodt H, Pinto JL (2002). Loss aversion and scale compatibility in two-attribute trade-offs. J. Math. Psychol..

[8] Attema AE, Brouwer WB, l’Haridon O, Pinto JL (2016). An elicitation of utility for quality of life under prospect theory. J. Health Econ..

[9] Stalmeier PF, Bezembinder TG (1999). The discrepancy between risky and riskless utilities: a matter of framing?. Med. Decis. Making.

[10] Pliskin JS, Shepard DS, Weinstein MC (1980). Utility functions for life years and health status. Oper. Res..

[11] Bleichrodt H, Schmidt U, Zank H (2009). Additive utility in prospect theory. Manag. Sci..

[12] Abdellaoui M, Bleichrodt H, L’Haridon O, van Dolder D (2016). Measuring loss aversion under ambiguity: a method to make prospect theory completely observable. J. Risk Uncertain.

[13] Abdellaoui M (2000). Parameter-free elicitation of utility and probability weighting functions. Manag. Sci..

[14] Miyamoto JM, Eraker SA (1989). Parametric models of the utility of survival duration: tests of axioms in a generic utility framework. Organ. Behav. Hum. Decis. Process..

[15] Herdman M, Gudex C, Lloyd A, Janssen M, Kind P, Parkin D, Bonsel G, Badia X (2011). Development and preliminary testing of the new five-level version of EQ-5D (EQ-5D-5L). Qual. Life Res..

[16] Wakker P, Deneffe D (1996). Eliciting von Neumann-Morgenstern utilities when probabilities are distorted or unknown. Manag. Sci..

[17] Cohen J (1988). Statistical Power Analysis for the Behavioral Sciences.

[18] Abellan-Perpinan JM, Pinto-Prades JL, Mendez-Martinez I, Badia-Llach X (2006). Towards a better QALY model. Health Econ..

[19] Bleichrodt H, Pinto JL (2005). The Validity of Qalys Under Non-expected Utility. Econ. J..

[20] Attema AE, Brouwer WB (2012). A test of independence of discounting from quality of life. J. Health Econ..

[21] Miyamoto JM, Eraker SA (1988). A multiplicative model of the utility of survival duration and health quality. J. Exp. Psychol. Gen..

[22] Attema AE, Bleichrodt H, L’Haridon O (2018). Ambiguity preferences for health. Health Econ..

[23] Bhatia S, Loomes G (2017). Noisy preferences in risky choice: a cautionary note. Psychol. Rev..

[24] Bleichrodt H, Pinto JL (2000). A parameter-free elicitation of the probability weighting function in medical decision analysis. Manag. Sci..

[25] Sutherland HJ, Llewellyn-Thomas H, Boyd NF, Till JE (1982). Attitudes toward quality of survival: the concept of “maximal endurable time”. Med. Decis. Mak..

[26] Versteegh MM, Vermeulen KM, Evers SM, de Wit GA, Prenger R, Stolk EA (2016). Dutch tariff for the five-level version of EQ-5D. Value Health.

[27] Pinto-Prades J-L, Abellan-Perpiñan J-M (2012). When normative and descriptive diverge: how to bridge the difference. Soc. Choice Welf.

[28] Bleichrodt H (2002). A new explanation for the difference between time trade-off utilities and standard gamble utilities. Health Econ..

[29] Drummond MF, Sculpher MJ, Claxton K, Stoddart GL, Torrance GW (2015). Methods for the Economic Evaluation of Health Care Programmes.

[30] Lipman, S., Brouwer, W., Attema, A.E.: Prospect theory and the corrective approach: policy implications of recent developments in QALY measurement. SSRN (2018). https://ssrn.com/abstract=3195710 10.1016/j.jval.2019.01.013 31277829

